# Evaluating the Role of Parental Education and Adolescent Health Problems in Educational Attainment

**DOI:** 10.1007/s13524-020-00919-y

**Published:** 2020-10-01

**Authors:** Janne Mikkonen, Hanna Remes, Heta Moustgaard, Pekka Martikainen

**Affiliations:** 1 Population Research Unit, Faculty of Social Sciences, University of Helsinki, PO Box 18 (Unioninkatu 35), FIN-00014, Helsinki, Finland; 2 Center for Health Equity Studies (CHESS), Department of Public Health Sciences, Stockholm University, Stockholm, Sweden; 3 The Max Planck Institute for Demographic Research, Rostock, Germany

**Keywords:** Education, Adolescent health, Health selection, Intergenerational transmission, Administrative data

## Abstract

This article reconsiders the role of social origin in health selection by examining whether parental education moderates the association between early health and educational attainment and whether health problems mediate the intergenerational transmission of education. We used longitudinal register data on Finns born in 1986–1991 (*n* = 352,899). We measured the completion of secondary and tertiary education until age 27 and used data on hospital care and medication reimbursements to assess chronic somatic conditions, frequent infections, and mental disorders at ages 10–16. We employed linear probability models to estimate the associations between different types of health problems and educational outcomes and to examine moderation by parental education, both overall in the population and comparing siblings with and without health problems. Finally, we performed a mediation analysis with g-computation to simulate whether a hypothetical eradication of health problems would weaken the association between parental and offspring education. All types of health problems reduced the likelihood of secondary education, but mental disorders were associated with the largest reductions. Among those with secondary education, there was further evidence of selection to tertiary education. High parental education buffered against the negative impact of mental disorders on completing secondary education but exacerbated it in the case of tertiary education. The simulated eradication of health problems slightly reduced disparities by parental education in secondary education (up to 10%) but increased disparities in tertiary education (up to 2%). Adolescent health problems and parental education are strong but chiefly independent predictors of educational attainment.

**Electronic supplementary material:**

The online version of this article (10.1007/s13524-020-00919-y) contains supplementary material, which is available to authorized users.

## Introduction

Higher education shows a positive impact on earnings, employment, life satisfaction, and workplace autonomy even in the context of educational expansion (Cairó and Cajner 2018; Edgerton et al. 2011; Psacharopoulos and Patrinos 2018). Among the hypothesized determinants of educational attainment, health problems in childhood and adolescence have been attracting growing attention—a trend most palpably demonstrated by the number of systematic reviews published around the same time (Eide and Showalter 2011; Esch et al. 2014; Hale et al. 2015; Melkevik et al. 2016; Suhrcke and de Paz Nieves 2011).

Children and adolescents experiencing poor health attain, on average, a lower education than their peers without health problems. This basic result seems to hold regardless of whether health problems are measured by self-rated health (Jackson 2009; Lê et al. 2013; Lynch and von Hippel 2016), specific health conditions and symptoms (Brekke 2015; Champaloux and Young 2015; Fletcher 2008, 2010; Kessler et al. 1995), or administrative records of diagnoses (Currie et al. 2010; Mikkonen et al. 2018; Roos et al. 2013), although comparable evidence suggests that the associations are generally the strongest for mental disorders and apply only to certain types of somatic conditions (Champaloux and Young 2015; Layte and McCrory 2013; Mikkonen et al. 2018; Uiters et al. 2014; van der Heide et al. 2016). Besides being of interest in its own right, the selection of healthier adolescents into higher levels of education also highlights the complexity of assessing whether education has benefits for adult health (Benson et al. 2018; von Hippel and Lynch 2014).

If given a causal interpretation, health-related selection to education may be explained by disengagement from the school environment and peers as a result of increased absenteeism and stigmatization (Basch 2011; Hale and Viner 2018; Needham 2009); delayed cognitive development (Bhutta et al. 2002; Boardman et al. 2002); reduced educational expectations held by parents, teachers, and subsequently the children and adolescents themselves (McLeod and Fettes 2007; Roeser et al. 1998); or compromised future orientation due to the reduced expected utility of pursuing a long-term time-consuming investment, such as higher education (Becker and Mulligan 1997; Haas et al. 2011). Among factors potentially confounding the relationship, parental education and other aspects of socioeconomic position—along with gender—have been the most common to adjust for in health selection research (Hale et al. 2015; Melkevik et al. 2016). A number of previous studies have even been able to fortify their inference with sibling fixed-effects models that control for all unobserved confounding shared by siblings (Currie et al. 2010; De Ridder et al. 2013; Evensen et al. 2016; Fletcher 2010; Fletcher and Wolfe 2008; Haas and Fosse 2008; Jackson 2009; Lê et al. 2013; Roos et al. 2013). However, something essential may be lost when social origin is treated as simply a nuisance factor because both the prevalence and experience of childhood health problems are likely to differ in advantaged as opposed to disadvantaged families (Case and Paxson 2006).

Our study reconsidered the role of social origin in health selection by examining whether parental education moderates the association between adolescent health problems and educational attainment, and whether health problems mediate the intergenerational transmission of educational attainment. Our investigation relied on Finnish longitudinal registry data, which enabled us to employ sibling fixed-effects models and follow educational attainment until age 27. Using administrative data from a Nordic welfare state, we were able to counterbalance the predominance of U.S.-based data, self-reported health measures, and short follow-ups of education in prior health selection research (Hale et al. 2015; Suhrcke and de Paz Nieves 2011). Given the small number of past studies examining our questions of interest with regard to parental education, our literature review considers investigations focusing on other aspects of parental socioeconomic position: namely, income and occupation.

### Moderation of Health Selection by Parental Education

Cumulative inequality theory emphasizes the cascading nature of adverse life experiences and the importance of resource availability and mobilization in shaping life trajectories (Ferraro and Shippee 2009; Schafer et al. 2011). The material, cultural, and social resources within a family may compensate for negative experiences in other domains of life (Parcel et al. 2010); conversely, adversities often become consequential only in the presence of other adversities or a shortage of compensatory resources (Schafer et al. 2011). Along these lines, the harmful impact of adolescent health problems could be diminished in families with high parental education and exacerbated in families with low-educated parents (Jackson 2015). Higher parental education has been shown to increase the time that parents spend actively engaged with their children (Craig 2006; Guryan et al. 2008) and to support children’s cognitive development (Lundborg et al. 2014; Noble et al. 2015).

At the same time, a contrary process has been acknowledged in the previous literature. Jackson (2009) noted that experiencing early health problems could be more harmful for adolescents coming from an advantageous background because the burden of health problems could deprive them of the very assets that set them in a favorable position, in terms of human capital accumulation. To put it differently, the expected educational attainment of this group of adolescents is higher; therefore, they have more to lose. Jackson (2009) found support for this latter theory, showing that the association between poor self-rated health and college attendance was weaker among adolescents belonging to an ethnic minority. These interactions were as strong among siblings as in the general population but not statistically significant, probably because of the small sample size. For parental education and income, the study showed similar, albeit nonsignificant, interactions.

Among other studies examining moderation by family resources, Flouri (2007) observed that hyperactivity at age 5 was more strongly associated with education at age 26 among children with higher maternal education. In contrast, Evensen et al. (2016) showed the association between adolescent mental health problems and years of completed education at age 27 to be equally strong in academic and nonacademic families, and similar evidence has been shown for low birth weight (Cheadle and Goosby 2010; Power et al. 2006). A later study by Jackson (2015) even found support for the idea that family social capital—as measured by time spent reading with children—ameliorates the negative impact of childhood health conditions on math and reading achievement test scores. In general, evidence on moderation is mixed, and there is an evident need for more research that would consider different types of health problems and long-term educational outcomes. Putting moderation to the test with a sibling comparison design might require a larger sample size than has been available previously (Evensen et al. 2016; Jackson 2009).

### Mediation of the Intergenerational Transmission of Education via Health Problems

Educational attainment is a social factor characterized by exceptionally strong intergenerational persistence with an average correlation of approximately .4 for parental and offspring years of schooling globally (Hertz et al. 2007). Simultaneously, vast evidence highlights education as a key socioeconomic determinant of health (Elo 2009; Mackenbach et al. 2015), and even early health is not allocated at random. Instead, there are noteworthy disparities in prenatal, child, and adolescent health and health behaviors according to parental education and other indicators of family socioeconomic circumstances (Currie 2011; Kramer et al. 2000; Reiss 2013; Viner et al. 2012). When combined with evidence on health-related selection to education, these associations suggest that early health could contribute to the intergenerational transmission of education.

Despite the cogent, much-discussed theoretical foundation (Case and Paxson 2006; Currie 2009; Palloni 2006), few studies have explicitly examined the importance of child or adolescent health for the intergenerational persistence of educational attainment or other aspects of socioeconomic position. Sznitman et al. (2017) used data from Norway and the United States to investigate whether adolescent self-rated health mediates the association of parental education and income with offspring high school completion: the mediated associations were statistically significant but covered less than 5% of the total effects in both countries. Similarly, indicators of prenatal and infant health seem to explain only a small part of the intergenerational transmission of education and income (Carvalho 2012) and occupational position (Härkönen et al. 2012). Despite positive associations between parental socioeconomic position and childhood health, Hoffmann et al. (2018) were unable to demonstrate mediation due to the absence of health selection effects. These results are in profound contrast to the study of Eriksson et al. (2005) in which a cluster of somatic and mental health problems, self-reported as lifetime measures at age 47, accounted for up to one-fourth of intergenerational income persistence in Denmark. However, retrospective reports covering several decades seem vulnerable to reverse causality and unobserved confounding.

Indirect evidence on the significance of health for intergenerational transmission is provided by older mobility studies showing that taller individuals are more likely to move upward and less likely to move downward (Blane et al. 2007; Cernerud 1995; Power et al. 2002). Along the same lines, Manor et al. (2003) discovered that poor self-rated health and absence from school because of ill health increased occupational downward mobility and reduced upward mobility in the United Kingdom, whereas Novak et al. (2012) did not observe similar associations for indices of chronic symptoms and psychological distress in Sweden. Overall, given the predominance of self-rated health, proxies of health status, and retrospective reports, earlier research provides a rather disjointed view of the issue. Moreover, to our knowledge, no previous intergenerational study has been able to use prospective longitudinal data to measure health problems in adolescence and educational attainment at an age high enough to reflect the completion of postsecondary education. In contrast to early childhood, adolescence is a time of decisive educational transitions, which could be seriously compromised by the experience of health problems.

### Current Study

Our primary goal is to examine the interconnections between adolescent health problems and parental education as predictors of educational attainment from two mutually non-exclusive perspectives: moderation by parental education and mediation by adolescent health problems. We argue that it is important to analyze these two phenomena in connection to each other because moderation by parental education could either amplify or attenuate mediation depending on whether high parental education buffers against or exacerbates the impact of health problems. Given the limited number of previous studies combining long follow-ups of completed education with sibling designs that control for confounders shared within a family, we started by assessing the basic associations between health problems and educational attainment. We separately analyzed selection to secondary education and selection to tertiary education—the latter only among those who attained secondary education, to distinguish it as a potentially separate phenomenon. As for selection to tertiary education, we also examined its independence of earlier selection to different tracks (general vs. vocational) in upper-secondary education.

We approached mediation from a policy-relevant perspective—in other words, by examining what would happen to the intergenerational transmission of education if adolescent health problems were, hypothetically, eradicated. Because we expected to show evidence of both social disparities in health and health-related selection to education, we hypothesized that the eradication of health problems would result in weaker associations between parental and offspring education. However, potential moderation by parental education could shape this basic pattern in an unexpected manner.

Considering that different types of health problems could be differently related to both social origin and educational outcomes, we further contributed to the previous literature by simultaneously examining chronic somatic conditions, frequent infections, and mental disorders. We expected these categories to capture different aspects of adolescent health, but we also considered their potential comorbidity.

We investigated the following questions:

1. Are different types of health problems in adolescence associated with educational attainment at age 27, both overall in the population and within sibships?
2. Does parental education moderate these associations?
3. Are health problems associated with tertiary education above and beyond selection to different tracks in upper-secondary education?
4. How large is the contribution of adolescent health problems to the association between parental and offspring education?

## Methods

### Data

We used registry-based data on all Finnish children born in 1986–1991 and living in mainland Finland at the end of 2000 (*n* = 382,518). Statistics Finland combined the required information from different registers with personal identification numbers, assigned to all residents in Finland and used in all registers (permission TK-53-525-11). Similar data were available on all known biological parents, who could be linked to their children with parental identification numbers included in the data. We received an anonymized data file through a remote-access system.

To increase the validity of our analysis, we excluded persons who did not live in Finland throughout the key measurement years, at ages 10–16 or at age 27 (*n* = 13,136). We further excluded persons who had received hospital treatment or lived in a care facility because of severe intellectual disabilities or persistent developmental disorders (*n* = 3,561) given that these persons less commonly pursue regular degree-level education. Moreover, we excluded persons whose biological mother or father was not in the data to be able to identify siblings (*n* = 8,044) and those who did not live with at least one biological parent at ages 10–14 (*n* = 9,494), thereby ensuring that our measures of parental education and other sociodemographic factors reflect the true childhood circumstances of the study population. The remaining analytical sample included 352,899 persons, of whom 163,430 had full siblings in the same sample. For these persons, we were able to achieve a complete linkage of education, health care, and sociodemographic data in the study period.

### Variables

#### Educational Attainment

Compulsory education in Finland lasts nine years and typically ends at age 16. After compulsory schooling, adolescents may complete a three-year upper-secondary degree in either a general or vocational track, both of which grant eligibility to apply to tertiary education (Pekkarinen et al. 2009). Although there are no dead-ends (i.e., degrees that do not make one eligible for higher-level studies) in the Finnish education system, pursuing tertiary education is more common for those completing the general track—as opposed to the vocational track—in upper-secondary education. In recent years, roughly 10% of Finnish young adults finished their educational career without completing a post-compulsory degree, and approximately 40% attained at least a lower tertiary degree, adding three to four years beyond upper-secondary education (Finnish National Agency for Education 2018). The fact that education is free of charge at all levels makes Finland an ideal context to study health-related selection to education.

Annual data on educational attainment, derived from the Register of Completed Education and Degrees, was available for years 1987–2018. We created two dummy variables to measure the completion of (1) at least upper-secondary and (2) at least tertiary education by age 27, which was the latest available age for the whole study population. When analyzing tertiary education, we included only those who had completed upper-secondary education (*n* = 263,239). For the analyses on moderation by parental education and mediation by health, we created a three-level (basic/secondary/tertiary) variable to measure the highest parental education among biological parents (i.e., either mother’s or father’s education) at age 14. Because of our sibling design, we further matched parental education within sibships to reflect the level of education observed when the youngest sibling was age 14.

#### Health Problems

We formed three combination indicators to reflect the presence of chronic somatic conditions, frequent infections, and mental disorders at ages 10–16. We chose this age range because it preceded upper-secondary education and because complete data on health care were available only for 1998 onward. The indicators of somatic conditions and mental disorders included data on visits to inpatient hospital care and outpatient specialized services provided by the National Institute for Health and Welfare as well as data on special reimbursement rights for long-term medication delivered by the Finnish Social Insurance Institution. Conditions were identified based on ICD-10 (International Classification of Diseases, 10th revision) codes in the inpatient/outpatient data (World Health Organization 2004) and reimbursement codes in the medication data, issued by medical specialists.

Our indicator of chronic somatic conditions (hereafter referred to as “somatic conditions”) covered 10 conditions that have shown negative associations with educational outcomes in previous studies (Benson et al. 2018; Champaloux and Young 2015; Mikkonen et al. 2018; Persson et al. 2013; Theodosiou et al. 2019; von Hippel and Lynch 2014): type 1 diabetes (ICD-10 code E10), epilepsy (G40-G41), cancer (C00-C97), dorsopathy/spinal disease (M40-M54), migraine and other headache syndromes (G43-G44), congenital heart disease (Q20-Q28), atopic dermatitis (L20), visual or hearing impairment (H54, H90-H91), rheumatoid arthritis (M05-M06, M08), and obesity (E66). For type 1 diabetes, epilepsy, rheumatoid arthritis, and cancer, we also included the corresponding special reimbursement right for long-term medication.

The group of mental disorders covered substance abuse (F10-F19), psychotic disorders (F20-F29), mood disorders (F30-F39), anxiety (F40-F42), stress-related and somatoform disorders (F43-F48), eating disorders and other behavioral syndromes associated with physiological disturbances (F50-F59), personality disorders (F60-F69), hyperkinetic and conduct disorders (F90-F91), other disorders with onset usually occurring in childhood and adolescence (F93-F98), and intentional self-harm (X71-X83). In addition to visits to inpatient and outpatient care, we included special reimbursement rights for psychosis medication. We did not include learning difficulties because they seemed susceptible to reverse causality (poor school performance promoting diagnosis).

For frequent infections, we exploited data on prescription drug purchases provided by the Finnish Social Insurance Institution. Anatomic Therapeutic Chemical (ATC) codes were used to identify antimicrobial drug purchases (ATC codes: J01, J02, J04, J05, P01, P02). Frequent infections were defined as having at least seven drug purchases at ages 10–16 (i.e., on average, one purchase per year); however, in the mediation analysis, we treated the total number of purchases as a continuous variable.

#### Control Variables

We adjusted for birth year and sex to account for cohort-specific differences in educational outcomes and health trends between adolescent girls and boys. As indicators of resource availability within a family, we controlled for family type (two parents/single-parent mother/single-parent father) and sibship size. Moreover, we adjusted for maternal age (with a linear and squared term) because low maternal age has been a risk factor of weaker psychosocial and educational outcomes in previous studies (Fergusson and Woodward 1999). Finally, to account for potential differences in access to treatment and educational opportunities, we adjusted for mother tongue (Finnish/Swedish/other), the region of residence (*n* = 18), and geographical urban–rural classification (a 7-point scale from inner-urban areas to sparsely populated rural areas, created by the Finnish Environmental Institute). Family type, sibship size, and regional background factors were measured as modes at ages 10–14 to control for annual fluctuations. The main effects analyses were also adjusted for parental education (see the earlier section Educational Attainment).

### Analysis

Our analytical procedure consisted of four parts, each designed to shed light on one of the study questions. First, we fitted linear probability models to estimate pairwise associations between the three health problem indicators and the two educational outcomes. These analyses were conducted in five steps. The first three steps progressively adjusted for birth year and sex, all control variables, and other types of health problems (i.e., the three types of health problems were included simultaneously); the last two steps employed fixed effects to estimate the associations within sibships, controlling for birth year and sex, and finally, for other types of health problems. Second, we included an interaction term between the different types of health problems and parental education to observe whether the associations differed according to parental education. This analysis was first adjusted for all control variables and subsequently conducted within sibships. Third, we reestimated the basic models for tertiary education while adjusting for track choice in upper-secondary education. Because the sibling comparison analyses required within-sibship variation in health problems, their effective sample sizes (reported in Table A1 in the online appendix) were much smaller than in the adjusted analyses. We estimated the linear probability models with Stata, version 15.1 (StataCorp 2017).

Last, we conducted a counterfactual-based mediation analysis to estimate the contribution of adolescent health problems to the association between parental and offspring education. Here, we combined the potential outcomes framework with g-computation to decompose the *total effect* of parental education into the *controlled direct effect* (i.e., the part remaining following a hypothetical eradication of health problems) and the *portion eliminated* (Wang and Arah 2015). Several accessible introductions to g-computation have been published (Snowden et al. 2011; Wang and Arah 2015), and in a simple setting such as ours, it is reminiscent of direct standardization. The same procedure was conducted for both secondary and tertiary education.

We fitted a logistic regression model to estimate logit[*E*(*Y* | *X*, **M**, **Z**)], where *Y* denotes secondary/tertiary education at age 27 (no/yes), *X* is parental education, **M** is a vector of mediators (somatic conditions, mental disorders, and the total count of antimicrobial drug purchases at ages 10–16), and **Z** is a vector of confounders. We allowed the health problems to interact with parental education. We used the parameters of this model to predict the probability of secondary/tertiary education at age 27 for every individual in the sample, averaged out the population-level probabilities for each level of parental education, and calculated the total effects (TE) of secondary and tertiary parental education compared with basic education:

$$ {\mathrm{TE}}_{\mathrm{secondary}}=\mathrm{E}\left[Y\left(X=\mathrm{secondary}\right)\hbox{--} Y\left(X=\mathrm{basic}\right)\right] $$

$$ {\mathrm{TE}}_{\mathrm{tertiary}}=\mathrm{E}\left[Y\left(X=\mathrm{tertiary}\right)\hbox{--} Y\left(X=\mathrm{basic}\right)\right]. $$

For controlled direct effects (CDE), we set all health problem indicators to 0 for everyone before predicting the probability of secondary/tertiary education at age 27. Thus, the controlled direct effects were calculated as follows:

$$ {\mathrm{CDE}}_{\mathrm{secondary}}=\mathrm{E}\left[Y\left(X=\mathrm{secondary},\mathbf{M}=0\right)\hbox{--} Y\left(X=\mathrm{basic},\mathbf{M}=0\right)\right] $$

$$ {\mathrm{CDE}}_{\mathrm{tertiary}}=\mathrm{E}\left[Y\left(X=\mathrm{tertiary},\mathbf{M}=0\right)\hbox{--} Y\left(X=\mathrm{basic},\mathbf{M}=0\right)\right]. $$

Portions eliminated were calculated by subtracting the controlled direct effect from the total effect. Finally, we produced bootstrapped 95% confidence intervals at the 2.5th and 97.5th percentiles by resampling the data 1,000 times with replacement. The whole procedure was run in R, version 3.5.1 (R Core Team 2018).

## Results

Our descriptive analysis showed that adolescents with somatic conditions, frequent infections, or mental disorders at ages 10–16 had attained secondary or tertiary education less often by age 27 (Table 1). When adjusted for birth year and sex, all coefficients clearly departed from 0 (Table 2): mental disorders were associated with the largest reduction in secondary education, by –19.6 percentage points (95% confidence interval = –20.2, –18.9); somatic conditions reduced its likelihood by –2.8 (–3.2, –2.4) and frequent infections by –1.4 percentage points (95% confidence interval = –1.7, –1.0). In comparison, the association of mental disorders with tertiary education was slightly weaker, and the association of somatic conditions was stronger. As for both secondary and tertiary education, subsequent adjustment for other control variables slightly weakened the coefficient of mental disorders. When the three types of health problems were included in the model simultaneously, the independent associations of somatic conditions and frequent infections became somewhat weaker. Within sibships, the associations of mental disorders were 4–5 percentage points weaker than in the total population, whereas the associations of frequent infections were up to 1.5 percentage points stronger.

**Table 1:** Distribution of the study population and the prevalence (%) of secondary and tertiary education at age 27 by background factors

	Full Sample (*n* = 352,899)			Sibling Sample (*n* = 163,430)		
		Education at Age 27 at Least (%)			Education at Age 27 at Least (%)	
Covariate^a^	%^d^	Secondary	Tertiary	%^d^	Secondary	Tertiary
Somatic Condition						
No	91.9	90.4	35.0	92.2	90.9	35.3
Yes	8.1	87.9	31.2	7.8	88.6	32.0
Frequent Infections						
No	90.9	90.3	34.8	91.8	90.8	35.1
Yes	9.1	89.0	33.6	8.2	89.6	34.5
Mental Disorder						
No	94.3	91.3	35.7	94.8	91.7	36.1
Yes	5.7	72.1	17.1	5.2	73.0	17.3
Sex						
Male	51.1	88.1	26.8	51.1	88.6	27.1
Female	48.9	92.3	42.9	48.9	92.8	43.4
Birth Year						
1986	16.2	89.1	35.0	15.3	90.1	36.9
1987	15.9	90.0	35.4	15.3	90.8	36.8
1988	16.8	89.8	34.9	18.0	90.3	35.5
1989	16.8	90.3	34.6	18.4	90.7	35.0
1990	17.2	90.7	34.0	16.7	90.8	33.5
1991	17.1	91.1	34.1	16.3	91.4	33.0
Maternal Age (continuous)	29.0			28.4		
	(5.2)			(4.7)		
Mother Tongue						
Finnish	93.9	90.2	34.4	94.0	90.8	34.9
Swedish	4.8	93.5	44.0	4.7	93.9	43.9
Other	1.4	76.0	21.7	1.3	74.1	17.6
Parental Education						
Tertiary	50.4	94.6	46.9	52.6	94.8	47.0
Secondary	42.8	87.2	23.5	41.9	87.7	23.1
Basic	6.8	76.0	14.1	5.6	74.3	12.3
Family Type^b^						
Two parents	82.3	91.8	37.0	85.0	92.1	37.1
Mother only	15.3	83.0	24.2	12.8	83.2	24.1
Father only	2.4	81.6	21.8	2.2	81.4	22.4
Sibship Size (continuous)^b^	2.8			3.3		
	(1.5)			(1.8)		
Urban-Rural Classification^b,c^						
Inner-urban	18.9	88.0	36.6	16.7	88.3	37.5
Outer-urban	29.5	90.0	36.1	28.5	90.5	37.0
Peri-urban	12.8	90.8	33.9	13.6	91.3	34.2
Local centers in rural areas	6.2	90.8	35.3	5.9	91.1	35.8
Rural areas close to urban	9.2	90.2	31.4	10.0	90.8	31.9
Rural heartland	15.0	91.7	34.1	16.0	92.2	34.4
Sparsely populated rural	8.4	91.2	30.4	9.3	91.7	30.5
Total	100.0	90.2	34.7	100.0	90.7	35.1

^a^Region of residence (mode at ages 10–14) is not shown because of the large number of categories (*n* = 18).

^b^Mode at ages 10–14.

^c^A nationwide geographical classification system by the Finnish Environmental Institute.

^d^The column presents means and standard deviations (shown in parentheses) for continuous variables.

**Table 2:** Difference in the probability of secondary and tertiary education at age 27 by the presence of health problems at ages 10–16, with 95% confidence intervals shown in parentheses

Health Problem	Model 1	Model 2	Model 3	Model 4	Model 5
A, Secondary Education					
Somatic condition	–.028	–.026	–.015	–.022	–.016
	(–.032, –.024)	(–.030, –.022)	(–.019, –.011)	(–.029, –.015)	(–.023, –.009)
Frequent infections	–.014	–.016	–.009	–.023	–.020
	(–.017, –.010)	(–.020, –.013)	(–.012, –.006)	(–.031, –.016)	(–0.027, –.013)
Mental disorder	–.196	–.171	–.170	–.119	–.117
	(–.202, –.189)	(–.177, –.165)	(–.176, –.163)	(–.130, –.107)	(–.129, –.106)
Birth year and sex	Yes	Yes	Yes	Yes	Yes
Other control variables^a^	No	Yes	Yes	No	No
Mutually adjusted^b^	No	No	Yes	No	Yes
Sibling fixed effects	No	No	No	Yes	Yes
*N*	352,899	352,899	352,899	163,430	163,430
B. Tertiary Education (among those with completed secondary education)					
Somatic condition	–.040	–.035	–.027	–.010	–.005
	(–.046, –.034)	(–.041, –.029)	(–.033, –.022)	(–.021, .002)	(–.017, .006)
Frequent infections	–.013	–.013	–.007	–.026	–.023
	(–.019, –.007)	(–.019, –.008)	(–.013, –.001)	(–.038, –.013)	(–.035, –.011)
Mental disorder	–.169	–.147	–.145	–.107	–.106
	(–.176, –.162)	(–.154, –.140)	(–.152, –.138)	(–.123, –.092)	(–.122, –.091)
Birth year and sex	Yes	Yes	Yes	Yes	Yes
Other control variables^a^	No	Yes	Yes	No	No
Mutually adjusted^b^	No	No	Yes	No	Yes
Sibling fixed effects	No	No	No	Yes	Yes
*N*	318,202	318,202	318,202	138,852	138,852

^a^The other control variables are maternal age, mother tongue, parental education, family type, sibship size, region, and urban-rural classification.

^b^Somatic conditions, frequent infections, and mental disorders were included in the model simultaneously.

**Fig. 1:**
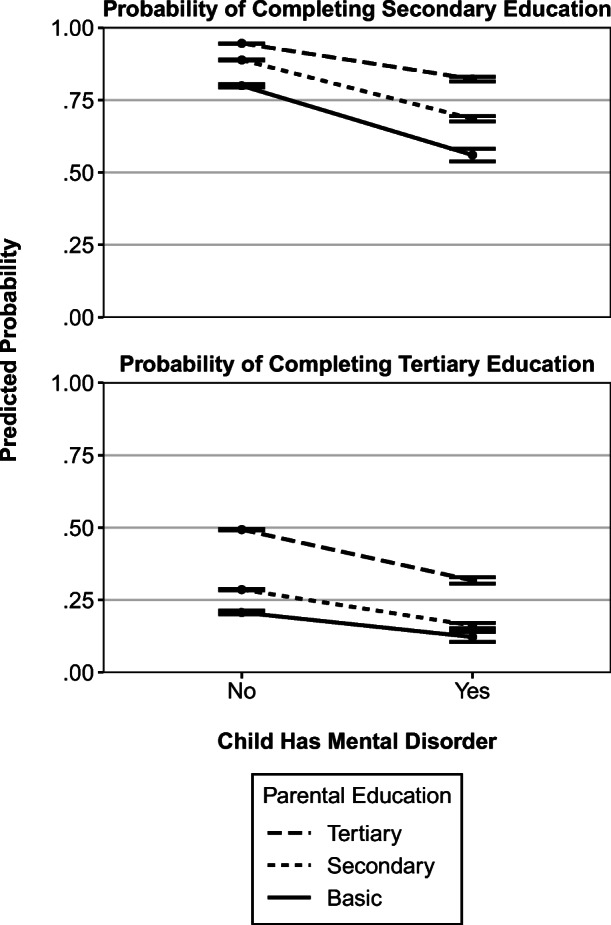
Predicted probabilities of secondary education (*n* = 352,899) and tertiary education (among those with completed secondary education; *n* = 318,202) at age 27, by parental education and the presence of mental disorders at ages 10–16. Data are adjusted for all control variables.

Mental disorders were less firmly associated with secondary education among adolescents with tertiary parental education than among others (Table 3, *p* < .001 overall and *p* = .001 within sibships). Conversely, higher parental education augmented the association between mental disorders and tertiary education (*p* < .001 overall and *p* = .017 within sibships). Figure 1 illustrates these interactions as predicted probabilities. Somatic conditions and frequent infections did not show systematic evidence of moderation by parental education. All coefficients (main effects and interaction terms) for the moderation analyses are presented in Tables A2–A5 in the online appendix. The results remained substantially identical when we separately examined moderation by paternal education and maternal education (not shown).

**Table 3:** Difference in the probability of secondary and tertiary education at age 27 by the presence of health problems at ages 10–16, by parental education, with *p* values testing the equivalence of coefficients and with 95% confidence intervals shown in parentheses

Health Problem	Parental Education	Overall^a^	Within Sibships^b^
A Secondary Education			
Somatic condition	Tertiary	–.024	–.018
		(–.028, –.019)	(–.026, –.009)
	Secondary	–0.029	–.030
		(–.035, –.022)	(–.042, –.018)
	Basic	–.023	.003
		(–.043, –.004)	(–.036, .041)
		*p* = .447	*p* = .121
Frequent infections	Tertiary	–.015	–.021
		(–.019, –.011)	(–.029, –.012)
	Secondary	–.016	–.029
		(–.022, –.010)	(–.041, –.017)
	Basic	–.025	–.003
		(–.043, –.006)	(–.045, .038)
		*p* = .614	*p* = .332
Mental disorder	Tertiary	–.123	–.096
		(–.131, –.115)	(–.110, –.081)
	Secondary	–.203	–.139
		(–.213, –.194)	(–.158, –.121)
	Basic	–.239	–.138
		(–.262, –.217)	(–.186, –.090)
		*p* < .001	*p* = .001
*N*		352,899	163,430
B. Tertiary Education (among those with completed secondary education)			
Somatic condition	Tertiary	–.041	–.011
		(–.049, –.032)	(–.028, .005)
	Secondary	–.028	–.003
		(–.037, –.020)	(–.020, .014)
	Basic	–.035	–.055
		(–.055, –,016)	(–.102, –.007)
		*p* = .135	*p* = .124
Frequent infections	Tertiary	–.013	–.017
		(–.021, –.004)	(–.035, .000)
	Secondary	–.015	–.039
		(–.027, –.006)	(–.057, –.021)
	Basic	–.010	–.002
		(–.030, .009)	(–.051, .047)
		*p* = .900	*p* = .149
Mental disorder	Tertiary	–.176	–.127
		(–.187, –.165)	(–.149, –.104)
	Secondary	–.124	–.086
		(–.133, –.115)	(–.108, –.065)
	Basic	–.085	–.069
		(–.103, –.066)	(–.123, –.016)
		*p* < .001	*p* = .017
*N*		318,202	138,852

^a^Adjusted for birth year, sex, maternal age, mother tongue, family type, sibship size, region, and urban-rural classification.

^b^Adjusted for birth year and sex.

To gain deeper insight on the mechanisms of selection to tertiary education, we examined the extent to which tertiary education was preceded by selection to different tracks (general vs. vocational) in upper-secondary education. When adjusted for upper-secondary track choice, the original associations between mental disorders and tertiary education became 40% to 60% weaker, somatic conditions showed an association only in the adjusted models, and frequent infections showed an association in the fixed-effects models (Table 4). Differences in upper-secondary track choice also proved important in explaining the observation that high parental education accentuates the impact of mental disorders on tertiary education. Adolescents experiencing health problems more often chose the vocational track, and this increase was larger among adolescents with high parental education (Fig. A1, online appendix). Moreover, when the predicted probabilities of tertiary education were calculated separately according to track choice, the stronger impact of mental disorders among the offspring of high-educated parents was present only among those completing the vocational track (Fig. A2, online appendix). This pattern seems to be due to the low probabilities (less than 10%) of continuing from the vocational track to tertiary education among adolescents with basic or secondary parental education, regardless of health.

**Table 4:** Difference in the probability of tertiary education (among those with completed secondary education) at age 27 by the presence of health problems at ages 10–16, adjusting for upper-secondary track choice (general vs. vocational), with 95% confidence intervals shown in parentheses

Health Problem	Model 1	Model 2	Model 3	Model 4	Model 5
Somatic Condition	–.011	–.012	–.010	.003	.005
	(–.016, –.006)	(–.017, –.007)	(–.014, –.004)	(–.008, .013)	(–.006, .016)
Frequent Infections	.002	.000	.002	–.016	–.015
	(–.003, .007)	(–.005, .005)	(–.003, .007)	(–.027, –.004)	(–.026, –.003)
Mental Disorder	–.076	–.067	–.067	–.060	–.060
	(–.082, –.070)	(–.074, –.061)	(–.073, –.060)	(–.074, –.046)	(–.074, –.045)
Track Choice	Yes	Yes	Yes	Yes	Yes
Birth Year and Sex	Yes	Yes	Yes	Yes	Yes
Other Control Variables^a^	No	Yes	Yes	No	No
Mutually Adjusted^b^	No	No	Yes	No	Yes
Sibling Fixed Effects	No	No	No	Yes	Yes
*N*	318,202	318,202	318,202	138,852	138,852

^a^Other control variables are maternal age, mother tongue, parental education, family type, sibship size, region, and urban-rural classification.

^b^Somatic conditions, frequent infections, and mental disorders were included in the model simultaneously.

Our mediation analysis simulated the effect of a hypothetical eradication of somatic conditions, mental disorders, and frequent infections on the association between parental and offspring education (Table 5). This simulated intervention eliminated 1.9 percentage points (roughly 10%) of the original 18.6 percentage point difference between tertiary and basic parental education in the likelihood of completing a secondary education. In contrast, the offspring of tertiary-educated parents were 31.0 percentage points more likely to attain a tertiary education than those with basic parental education, and the eradication of health problems strengthened this association by 0.7 percentage points (approximately 2%). The original differences between secondary and basic parental education were smaller, but they exhibited similar relative changes. When we conducted the same decomposition by eradicating each type of health problems individually, both mental disorders and frequent infections contributed to the differences in secondary education, whereas the eradication of somatic conditions did not influence the association between parental and offspring education (Table A6 in the online appendix).

**Table 5:** Decomposition of the total effect of parental education on offspring secondary and tertiary education into a controlled direct effect and a portion eliminated, following a hypothetical eradication of adolescent health problems

Parental Education	Total Effect	Controlled Direct Effect	Portion Eliminated
A Secondary Education (*n* = 352,899)			
Basic (ref.)			
Secondary	.113	.101	.012
	(.107, .118)	(.094, .108)	(.007, .016)
Tertiary	.186	.167	.019
	(.180, .192)	(.160, .174)	(.015, .024)
B Tertiary Education (among those with completed secondary education; *n* = 318,202)			
Basic (ref.)			
Secondary	.083	.087	–.003
	(.076, .089)	(.078, .095)	(–.009, .002)
Tertiary	.310	.317	–.007
	(.303, .316)	(.309, .326)	(–.012, –.002)

As a sensitivity analysis, we examined whether our results applied equally to men and women. Although women were more likely to complete both secondary and tertiary education by age 27, only mental disorders differed by sex in the strength of health-related selection to education. Their association with secondary education was approximately 5 percentage points weaker for women (–0.147 vs. –0.201, *p* < .001), adjusting for other covariates, and the association between mental disorders and tertiary education was roughly 4 percentage points stronger (–0.164 vs. –0.123, *p* < .001). Despite this, parental education moderated the impact of mental disorders similarly among men and women (not shown). Table A7 in the online appendix reports the mediation analysis stratified by sex. Overall, the associations were quite similar among men and women. High parental education was more clearly associated with secondary education among men, but the hypothetical eradication of health problems eliminated a larger share of the original differences among women (up to 14% vs. up to 7%).

## Discussion

Using longitudinal register data with sibling fixed-effects models, the present investigation demonstrates that adolescent health problems may have a permanent negative impact on educational attainment regardless of parental education. These associations are most evident for mental disorders, but they also deserve attention in the case of chronic somatic conditions and frequent infections. High parental education seems to partially protect against not completing secondary education as a result of mental disorders, but this buffering does not extend to tertiary education. Mental disorders were instead associated with the largest absolute reductions in tertiary education among the offspring of highly educated parents. Our further analysis revealed that this pattern was largely explained by differences in track choice during upper-secondary education: mental disorders increased the likelihood of choosing a vocational track, and even more so among adolescents with highly educated parents. Although previous research has documented socioeconomic gradients in child and adolescent health (Reiss 2013; Viner et al. 2012), the role of these gradients as an explanation to the intergenerational transmission of education is meager (near 10%) and is limited to secondary education.

### Comparison With Other Studies

The results are in line with previous studies highlighting the particular significance of mental disorders (Currie et al. 2010; Layte and McCrory 2013; Mikkonen et al. 2018; Uiters et al. 2014; van der Heide et al. 2016). However, our study benefitted from an exceptionally long follow-up of educational outcomes until age 27 and the opportunity to analyze selection to tertiary education in addition to secondary education. Whereas impairments in short-term educational outcomes could be caused by delays in educational careers, our findings emphasize the permanence of health selection. Simultaneously, we demonstrated that early adolescent mental disorders show an association with tertiary education that is partly independent of selection processes during secondary education.

In line with a number of other studies employing sibling comparison designs to adjust for unobserved family-level confounding, our study endorses the conclusion that the associations are substantially similar within families, albeit slightly weaker, compared with estimates obtained in the total population (Currie et al. 2010; Fletcher 2010; Haas and Fosse 2008; Jackson 2009; Lê et al. 2013). In contrast to the results of De Ridder et al. (2013), the association of somatic conditions did not completely disappear in the fixed-effects analysis, perhaps because of our larger sample size and the inclusion of more severe health conditions. Small sample sizes have been a common problem in previous fixed-effects analyses (Fletcher 2010; Haas and Fosse 2008; Lê et al. 2013).

Mental disorders were more strongly associated with secondary education among the offspring of low-educated parents. This result is in line with Jackson (2015), who observed family social capital to buffer against the negative impact of childhood health conditions (physical and mental conditions combined) on math and reading test scores. Taken together, these results suggest that highly educated parents are able to compensate for offspring health problems at the early stages of education. However, we also showed that somatic conditions and frequent infections are equally associated with secondary education regardless of parental education.

When analyzing educational outcomes taking place after adolescence, previous studies have shown that an advantageous social background exacerbates the negative associations (Flouri 2007; Jackson 2009) or that no differences exist in the associations between academic and nonacademic families (Evensen et al. 2016). We also observed the negative impact of mental disorders on tertiary education to be the largest among the offspring of highly educated parents, but this pattern was no longer evident when analyzed separately according to upper-secondary track choice. Thus, it seems that although highly educated parents can protect against the noncompletion of secondary education resulting from mental disorders, health-related selection to tertiary education is nevertheless largely preceded by upper-secondary track choices even among adolescents with high parental education. Adolescents with health problems are more likely to choose a vocational track, which qualifies young people to work in manual or lower nonmanual jobs and thus less commonly leads to studies at the tertiary level. This increase in choosing a vocational track is even steeper among adolescents with high parental education, which explains why the association between mental disorders and tertiary education is the strongest in this group. The implications of adolescent health problems for study track choice should be investigated more thoroughly in future studies.

Sznitman et al. (2017) discovered that poor self-rated health mediates less than 5% of the association between parental education and high school completion. We strengthen this evidence by showing that roughly 10% of the differences in secondary education according to parental education can be explained by a cluster of serious and long-term health problems. Moreover, in the case of tertiary education, health problems seem to slightly equalize the differences, which could reflect the fact that mental disorders were more strongly associated with not completing tertiary education in families with high parental education.

Hoffmann et al. (2018) measured childhood health with a latent construct that was not based on specific diagnoses: they did not observe mediation because of no evidence of health-related selection to education. In our study, the minor contribution of health to the intergenerational transmission of education relates to the small prevalence of serious adolescent health problems and the unexpectedly weak associations between parental education and offspring health problems (presented in Table A8 in the online appendix). Altogether, this variation in results highlights the importance of the way health problems are measured. Future research aiming to identify the total contribution of “educationally relevant health disparities” (Basch 2011) would probably have to combine data on specific diagnoses with measurements of broader lifestyle-related factors, such as low physical activity.

### Strengths and Limitations

Compared with previous work, our study benefitted from a large, population-based data set, combining precise and objective measurements of both parental and offspring education with a seven-year follow-up of adolescent health problems. With registry data, we were able to avoid the typical pitfalls of longitudinal survey research: attrition, selective dropout, item nonresponse, and unreliable reporting. Moreover, the availability of education follow-up until the end of 2018 allowed us to provide information on the most recent birth cohorts—children born during 1986–1991—to finish their educational career. The fact that these are post-educational expansion cohorts in Finland (Statistics Finland 2018) is an additional advantage because it underpins the commensurability of parental and offspring education.

At the same time, the use of register data confined our measurement to health conditions that received treatment, which could introduce bias, particularly in the case of less severe mental disorders. Given that we were able to either control for the most likely regional and socioeconomic correlates of treatment-seeking or conduct the analyses within sibships, we have no reason to believe that our basic and moderation analyses suffered from major biases. However, the same does not necessarily apply to our mediation analysis, which is sensitive to the correct estimation of the prevalence of adolescent health problems according to parental education: if highly educated parents are more likely to seek treatment for their children with minor psychiatric problems, our analysis may have underestimated the contribution of health problems. Although we could not completely eliminate these concerns, the Finnish highly subsidized public health-care system and several extensive health checks during compulsory schooling (Ministry of Social Affairs and Health 2013), as well as the use of a seven-year age span for measurement, are all factors that mitigated socioeconomic differences in treatment and protected against both systematic and random bias in the detection of adolescent health problems. In a study from Finland, parental education did not predict seeking treatment for adolescent emotional and behavioral problems (Sourander et al. 2001). Similar results have been obtained in the Netherlands (Verhulst and van der Ende 1997).

Another point of concern relates to our measurement of offspring education at age 27, considering that the median age of first-time entrants to tertiary education in Finland is among the highest in Organisation for Economic Cooperation and Development countries (OECD 2018:198). As a sensitivity analysis, we reproduced the main effects analysis by assuming that enrolled students will eventually complete their studies (Table A9 in the online appendix). The associations between health problems and secondary education became slightly weaker, whereas the associations with tertiary education remained similar to those based on completed education. Although these results do not challenge our conclusions, they provide only a partial remedy because there could be health-related differences in the discontinuation of education. Besides gathering even longer follow-ups of education, future studies could extend our analysis on the key stages of selection by estimating whether early adolescent health has an independent contribution to long-term educational outcomes, regardless of later health problems. Early adolescent mental disorders are likely associated, to some extent, with long-term educational outcomes because of their recurrent nature.

Similar to previous studies, we were unable to control for unobserved confounders of the association between health problems and education that were not shared within the family. Specifically, evidence shows that part of the association between health and education is explained by a genetic overlap between health and education (Boardman et al. 2015), not fully accounted for by sibling fixed-effects models. It is worth noting that sibling comparisons rest on within-sibship variation in health problems and might therefore not be fully generalizable to only children or families where all children have encountered health problems. Additionally, sibling models could produce conservative estimates if one sibling’s health problems influence the education of other siblings without health problems. This could be the case if, for instance, behavioral problems affect other siblings’ educational outcomes via a disorganized home environment (Sjölander et al. 2016). With these limitations in mind, our sibling results are best seen as a useful further confirmation of our adjusted estimates.

### Conclusion

Our results suggest that adolescent health problems and parental education are strong but chiefly independent predictors of educational attainment and do not form a substantial causal chain stretching from low parental education to health problems and subsequently to lower educational attainment. Health problems nonetheless account for approximately 10% of the association between parental education and the completion of secondary education both because health problems are slightly more common among the offspring of low-educated parents and because high parental education seems to protect against the negative impact of mental disorders on secondary education. We further contribute to the literature by showing that health problems reduce the likelihood of completing tertiary education even among those who manage to attain secondary education. Nevertheless, considering that health-related differences in completing tertiary education are foreshadowed by upper-secondary track choices, supportive interventions are likely to make the largest difference in early adolescence.

## Electronic supplementary material

ESM 1 (PDF 254 kb)

## Data Availability

Because of data protection regulations, the data file used in this study can be accessed only through the remote access system of Statistics Finland. Data requests should be addressed to Statistics Finland. For more information, see http://www.stat.fi/tup/mikroaineistot/etakaytto_en.html.
