# Supplementary material for: Evaluating the Role of Parental Education and Adolescent Health Problems in Educational Attainment
Source: Demography. 2020 Oct 1;57(6):2245–67. doi: 10.1007/s13524-020-00919-y (PMC7732787; doi:10.1007/s13524-020-00919-y)

Table A1 Numbers of siblings discordant in health status according to parental education and the type of health problem

		Parental education		
Health problems	Overall	Tertiary	Secondary	Basic
Somatic condition	24,459	12,399	10,560	1,500
Frequent infections	21,863	11,098	9,527	1,238
Mental disorder	15,788	7,334	7,171	1,283

Table A2 Adjusted linear probability models (beta coefficients, b, and robust standard errors, se) for secondary education at age 27 with interactions between different types of health problems and parental education

	Somatic conditions		Frequent infections		Mental disorders	
Covariates[a]	b	se	b	se	b	se
Parental education (tertiary)						
Secondary	–.063***	(.001)	–.063***	(.001)	–.057***	(.001)
Basic	–.158***	(.003)	–.157***	(.003)	–.146***	(.003)
Somatic condition	–.024***	(.002)				
Secondary X Somatic condition	–.005	(.004)				
Basic X Somatic condition	.000	(.010)				
Frequent infections			–.015***	(.002)		
Secondary X Frequent infections			–.000	(.004)		
Basic X Frequent infections			–.009	(.010)		
Mental disorder					–.123***	(.004)
Secondary X Mental disorder					–.080***	(.006)
Basic X Mental disorder					–.116***	(.012)
Sex (male)						
Female	.042***	(.001)	.042***	(.001)	.044***	(.001)
Birth year (1986)						
1987	.008***	(.002)	.007***	(.002)	.008***	(.002)
1988	.006***	(.002)	.005**	(.002)	.008***	(.002)
1989	.010***	(.002)	.009***	(.002)	.011***	(.002)
1990	.013***	(.002)	.012***	(.002)	.015***	(.002)
1991	.016***	(.002)	.016***	(.002)	.019***	(.002)
Maternal age, linear	.028***	(.001)	.028***	(.001)	.026***	(.001)
Maternal age, squared	–.000***	(.000)	–.000***	(.000)	–.000***	(.000)
Mother tongue (Finnish)						
Swedish	.028***	(.002)	.028***	(.002)	.025***	(.002)
Other	–.059***	(.006)	–.059***	(.006)	–.060***	(.006)
Family type (two parents)						
Mother only	–.071***	(.002)	–.071***	(.002)	–.064***	(.002)
Father only	–.080***	(.004)	–.081***	(.004)	–.073***	(.004)
Sibship size	–.008***	(.000)	–.008***	(.000)	–.008***	(.000)
Urban–rural class. (inner-urban)						
Outer urban	.007***	(.002)	.007***	(.002)	.007***	(.002)
Peri-urban	.016***	(.002)	.016***	(.002)	.015***	(.002)
Local centers in rural areas	.014***	(.002)	.014***	(.002)	.013***	(.002)
Rural areas close to urban	.014***	(.002)	.014***	(.002)	.013***	(.002)
Rural heartland	.021***	(.002)	.021***	(.002)	.019***	(.002)
Sparsely populated rural	.024***	(.002)	.024***	(.002)	.021***	(.002)
Constant	.448***	(.016)	.448***	(.016)	.488***	(.016)
N	352,899		352,899		352,899	

* p < .05 ** p<.01 *** p<.001

[a]Reference category is shown in parentheses. Region of residence is omitted because of the large number of categories (*n* = 18).

Table A3 Adjusted linear probability models (beta coefficients, b, and robust standard errors, se) for tertiary education at age 27 with interactions between different types of health problems and parental education

	Somatic conditions		Frequent infections		Mental disorders	
Covariates[a]	b	se	b	se	b	se
Parental education (tertiary)						
Secondary	–.206***	(.002)	–.205***	(.002)	–.207***	(.002)
Basic	–.283***	(.003)	–.283***	(.003)	–.286***	(.003)
Somatic condition	–.041***	(.005)				
Secondary X Somatic condition	.012*	(.006)				
Basic X Somatic condition	.005	(.011)				
Frequent infections			–.013**	(.004)		
Secondary X Frequent infections			–.002	(.006)		
Basic X Frequent infections			.002	(.011)		
Mental disorder					–.176***	(.006)
Secondary X Mental disorder					.051***	(.007)
Basic X Mental disorder					.091***	(.011)
Sex (male)						
Female	.165***	(.002)	.164***	(.002)	.167***	(.002)
Birth year (1986)						
1987	–.003	(.003)	–.003	(.003)	–.003	(.003)
1988	–.010***	(.003)	–.010***	(.003)	–.009**	(.003)
1989	–.016***	(.003)	–.017***	(.003)	–.015***	(.003)
1990	–.025***	(.003)	–.026***	(.003)	–.024***	(.003)
1991	–.028***	(.003)	–.029***	(.003)	–.026***	(.003)
Maternal age, linear	.043***	(.001)	.043***	(.001)	.041***	(.001)
Maternal age, squared	–.001***	(.000)	–.001***	(.000)	–.001***	(.000)
Mother tongue (Finnish)						
Swedish	.071***	(.005)	.071***	(.005)	.069***	(.005)
Other	–.023**	(.008)	–.023**	(.008)	–.023**	(.008)
Family type (two parents)						
Mother only	–.096***	(.002)	–.097***	(.002)	–.092***	(.002)
Father only	–.092***	(.005)	–.092***	(.005)	–.088***	(.005)
Sibship size	–.016***	(.001)	–.016***	(.001)	–.016***	(.001)
Urban–rural class. (inner-urban)						
Outer urban	–.009***	(.003)	–.009**	(.003)	–.009***	(.003)
Peri-urban	–.026***	(.003)	–.026***	(.003)	–.026***	(.003)
Local centers in rural areas	–.009*	(.004)	–.009*	(.004)	–.010*	(.004)
Rural areas close to urban	–.034***	(.004)	–.034***	(.004)	–.034***	(.004)
Rural heartland	–.014***	(.003)	–.014***	(.003)	–.015***	(.003)
Sparsely populated rural	–.027***	(.004)	–.026***	(.004)	–.027***	(.004)
Constant	–.252***	(.022)	–.254***	(.022)	–.228***	(.022)
N	318,202		318,202		318,202	

* $p < .05$ ** $p<.01$ *** $p<.001$

[a]Reference category is shown in parentheses. Region of residence is omitted because of the large number of categories (*n* = 18).

Table A4 Sibling fixed-effects linear probability models (beta coefficients, b, and robust standard errors, se) for secondary education at age 27 with interactions between different types of health problems and parental education

	Somatic conditions		Frequent infections		Mental disorders	
Covariates[a]	b	se	b	se	b	se
Somatic condition (tertiary)	–.018***	(.004)				
Secondary X Somatic condition	–.012	(.007)				
Basic X Somatic condition	.020	(.020)				
Frequent infections (tertiary)			–.021***	(.004)		
Secondary X Frequent infections			–.009	(.007)		
Basic X Frequent infections			.017	(.022)		
Mental disorder (tertiary)					–.096***	(.007)
Secondary X Mental disorder					–.044***	(.012)
Basic X Mental disorder					–.042	(.025)
Sex (male)						
Female	.047***	(.002)	.047***	(.002)	.046***	(.002)
Birth year (1986)						
1987	.006*	(.003)	.007*	(.003)	.006	(.003)
1988	.001	(.003)	.002	(.003)	.000	(.003)
1989	.002	(.003)	.003	(.003)	.001	(.003)
1990	.002	(.003)	.004	(.003)	.001	(.003)
1991	.007*	(.003)	.008**	(.003)	.005	(.003)
Constant	.883***	(.002)	.886***	(.002)	.884***	(.002)
N	163,430		163,430		163,430	

* p < .05 ** p<.01 *** p<.001

[a]Reference category is shown in parentheses.

Table A5 Sibling fixed-effects linear probability models (beta coefficients, b, and robust standard errors, se) for tertiary education at age 27 with interactions between different types of health problems and parental education

	Somatic conditions		Frequent infections		Mental disorders	
Covariates[a]	b	se	b	se	b	se
Somatic condition (tertiary)	–.011	(.008)				
Secondary X Somatic condition	.009	(.012)				
Basic X Somatic condition	–.043	(.026)				
Frequent infections (tertiary)			–.017	(.009)		
Secondary X Frequent infections			–.021	(.013)		
Basic X Frequent infections			.016	(.027)		
Mental disorder (tertiary)					–.127***	(.011)
Secondary X Mental disorder					.040*	(.016)
Basic X Mental disorder					.057	(.030)
Sex (male)						
Female	.171***	(.003)	.171***	(.003)	.172***	(.003)
Birth year (1986)						
1987	–.010	(.006)	–.010	(.006)	–.010	(.006)
1988	–.029***	(.005)	–.030***	(.005)	–.028***	(.005)
1989	–.035***	(.005)	–.036***	(.005)	–.034***	(.005)
1990	–.056***	(.005)	–.057***	(.005)	–.054***	(.005)
1991	–.065***	(.005)	–.066***	(.005)	–.064***	(.005)
Constant	.349***	(.004)	.351***	(.004)	.351***	(.004)
N	138,852		138,852		138,852	

* p < .05 ** p<.01 *** p<.001
[a]Reference category is shown in parentheses.

Table A6 Decomposition of the total effect of parental education on offspring secondary (n = 352,899) and tertiary (n = 318,202) education into a controlled direct effect and a portion eliminated, following a hypothetical eradication of somatic conditions, frequent infections, or mental disorders

I) Somatic conditions

A. Secondary education

Parental education	Total effect	Controlled direct effect	Portion eliminated
Basic (ref.)			
Secondary	.113 (.107, .119)	.113 (.107, .119)	–.001 (–.002, .002)
Tertiary	.186 (.180, .192)	.185 (.179, .192)	.001 (–.001, .002)

B. Tertiary education (among those with completed secondary education)

Parental education	Total effect	Controlled direct effect	Portion eliminated
Basic (ref.)			
Secondary	.083 (.076, .089)	.082 (.075, .089)	.001 (–.001, .002)
Tertiary	.310 (.304, .316)	.310 (.304, .317)	.000 (–.002, .001)

II) Frequent infections

A. Secondary education

Parental education	Total effect	Controlled direct effect	Portion eliminated
Basic (ref.)			
Secondary	.113 (.107, .119)	.106 (.099, .113)	.007 (.002, .011)
Tertiary	.186 (.180, .192)	.177 (.170, .184)	.009 (.005, .013)

B. Tertiary education (among those with completed secondary education)

Parental education	Total effect	Controlled direct effect	Portion eliminated
Basic (ref.)			
Secondary	.083 (.076, .089)	.086 (.078, .094)	–.003 (–.008, .001)
Tertiary	.310 (.304, .316)	.315 (.307, .324)	–.005 (–.010, .000)

III) Mental disorders

A. Secondary education

Parental education	Total effect	Controlled direct effect	Portion eliminated
Basic (ref.)			
Secondary	.113 (.107, .119)	.107 (.101, .113)	.006 (.004, .008)
Tertiary	.186 (.180, .192)	.174 (.169, .180)	.012 (.010, .014)

B. Tertiary education (among those with completed secondary education)

Parental education	Total effect	Controlled direct effect	Portion eliminated
Basic (ref.)			
Secondary	.083 (.076, .089)	.084 (.077, .090)	–.001 (–.002, .000)
Tertiary	.310 (.304, .316)	.313 (.306, .319)	–.003 (–.004, –.001)

Table A7 Sex-specific decomposition of the total effect of parental education on offspring secondary and tertiary education into a controlled direct effect and a portion eliminated, following a hypothetical eradication of health problems

I) Men

A. Secondary education (n = 180,348)

Parental education	Total effect	Controlled direct effect	Portion eliminated
Basic (ref.)			
Secondary	.121 (.113, .129)	.113 (.103, .124)	.007 (.001, .015)
Tertiary	.205 (.197, .213)	.192 (.182, .202)	.015 (.010, .020)

B. Tertiary education (among those with completed secondary education; n = 158,913)

Parental education	Total effect	Controlled direct effect	Portion eliminated
Basic (ref.)			
Secondary	.071 (.063, .078)	.075 (.065, .085)	–.004 (–.010, .002)
Tertiary	.288 (.280, .295)	.296 (.287, .306)	–.008 (–.015, –.003)

II) Women

A. Secondary education (n = 172,551)

Parental education	Total effect	Controlled direct effect	Portion eliminated
Basic (ref.)			
Secondary	.104 (.097, .112)	.090 (.081, .100)	.014 (.008, .020)
Tertiary	.166 (.158, .174)	.143 (.134, .151)	.024 (.018, .029)

B. Tertiary education (among those with completed secondary education; n = 159,289)

Parental education	Total effect	Controlled direct effect	Portion eliminated
Basic (ref.)			
Secondary	.095 (.085, .104)	.097 (.084, .110)	–.002 (–.010, .006)
Tertiary	.335 (.326, .344)	.340 (.326, .352)	–.005 (–.013, .004)

Table A8 Prevalence of somatic conditions, frequent infections, and mental disorders (%) at ages 10–16 by parental education (n = 352,899)

Parental education	Somatic	Infection	Mental
Tertiary	7.7	8.7	4.9
Secondary	8.4	9.5	6.2
Basic	8.7	9.6	8.2
P-value[a]	p<.001	p<.001	p<.001

[a]Chi-squared tests test the equality of prevalence.

Table A9 Difference in the probability of secondary and tertiary education at age 27 by the presence of health problems at ages 10–16, assuming students will eventually complete the degree they are pursuing, with 95% confidence intervals shown in parentheses

A. Secondary education

Health problem	Model 1	Model 2	Model 3	Model 4	Model 5
Somatic condition	–.024	–.022	–.014	–.018	–.013
	(–.028, –.021)	(–.026, –.019)	(–.018, –.011)	(–.025, –.012)	(–.020, –.007)
Frequent infections	–.012	–.014	–.008	–.020	–.017
	(–.017, –.009)	(–.017, –.011)	(–.011, –.005)	(–.027, –.014)	(–.024, –.011)
Mental disorder	–.154	–.134	–.133	–.098	–.096
	(–.160, –.148)	(–.140, –.129)	(–.138, –.127)	(–.108, –.087)	(–.107, –.086)
Birth year and sex	Yes	Yes	Yes	Yes	Yes
Other control variables[a]	No	Yes	Yes	No	No
Mutually adjusted	No	No	Yes	No	Yes
Sibling fixed effects	No	No	No	Yes	Yes
N	352,899	352,899	352,899	163,430	163,430

B. Tertiary education (among those with completed secondary education)

Health problem	Model 1	Model 2	Model 3	Model 4	Model 5
Somatic condition	–.044	–.037	–.030	–.021	–.017
	(–.050, –.038)	(–.043, –.031)	(–.036, –.024)	(–.033, –.009)	(–.029, –.005)
Frequent infections	–.015	–.013	–.006	–.016	–.013
	(–.021, –.009)	(–.019, –.007)	(–.012, –.001)	(–.029, –.004)	(–.026, –.001)
Mental disorder	–.163	–.144	–.141	–.102	–.101
	(–.171, –.155)	(–.151, –.136)	(–.148, –.133)	(–.118, –.086)	(–.116, –.085)
Birth year and sex	Yes	Yes	Yes	Yes	Yes
Other control variables[a]	No	Yes	Yes	No	No
Mutually adjusted[b]	No	No	Yes	No	Yes
Sibling fixed effects	No	No	No	Yes	Yes
N	318,202	318,202	318,202	138,852	138,852

[a]Maternal age, mother tongue, parental education, family type, sibship size, region, urban–rural classification.
[b]Somatic conditions, frequent infections, and mental disorders were included in the model simultaneously.

Fig A1 Predicted probabilities of completing the general track (instead of the vocational track) among those who completed upper-secondary education by age 27 (n=318,202) by parental education and the presence of mental disorders at ages 10-16. Data are adjusted for all control variables. Statistical significance of the interaction between parental education and mental disorders: p<.001

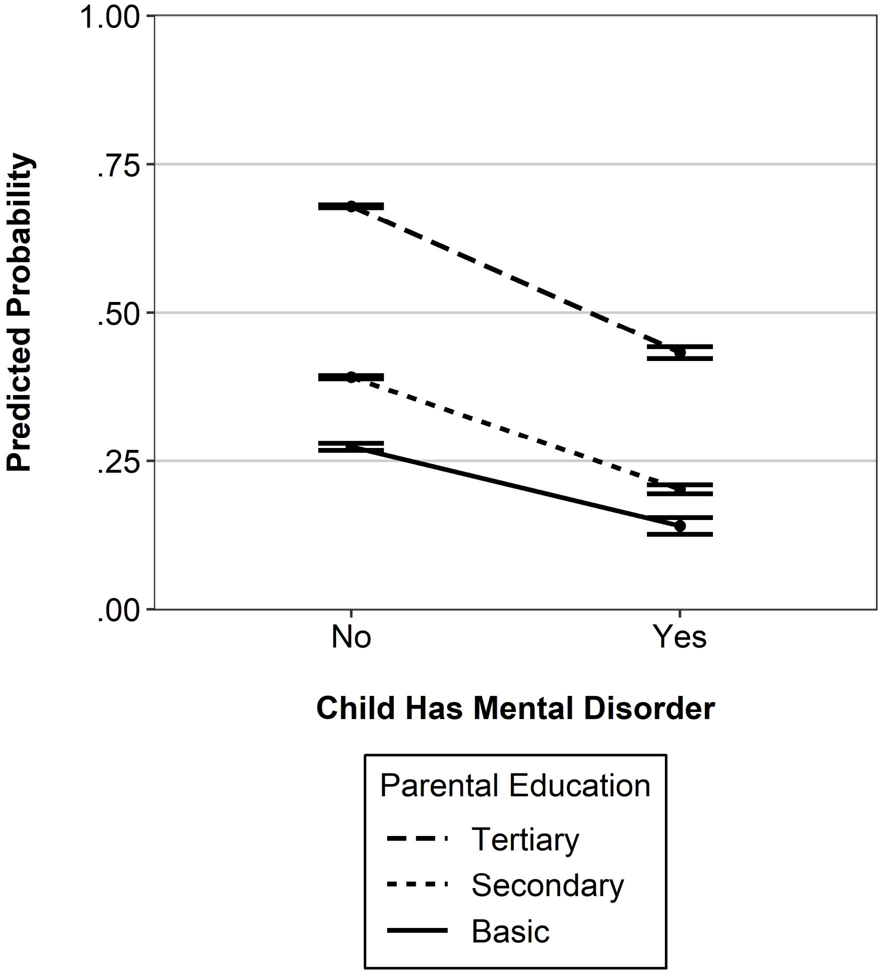

Fig A2 Predicted probabilities of tertiary education (among those with completed secondary education, n=318,202) at age 27 by parental education, upper-secondary track-choice (general vs. vocational), and the presence of mental disorders at ages 10–16. Data are adjusted for all control variables.

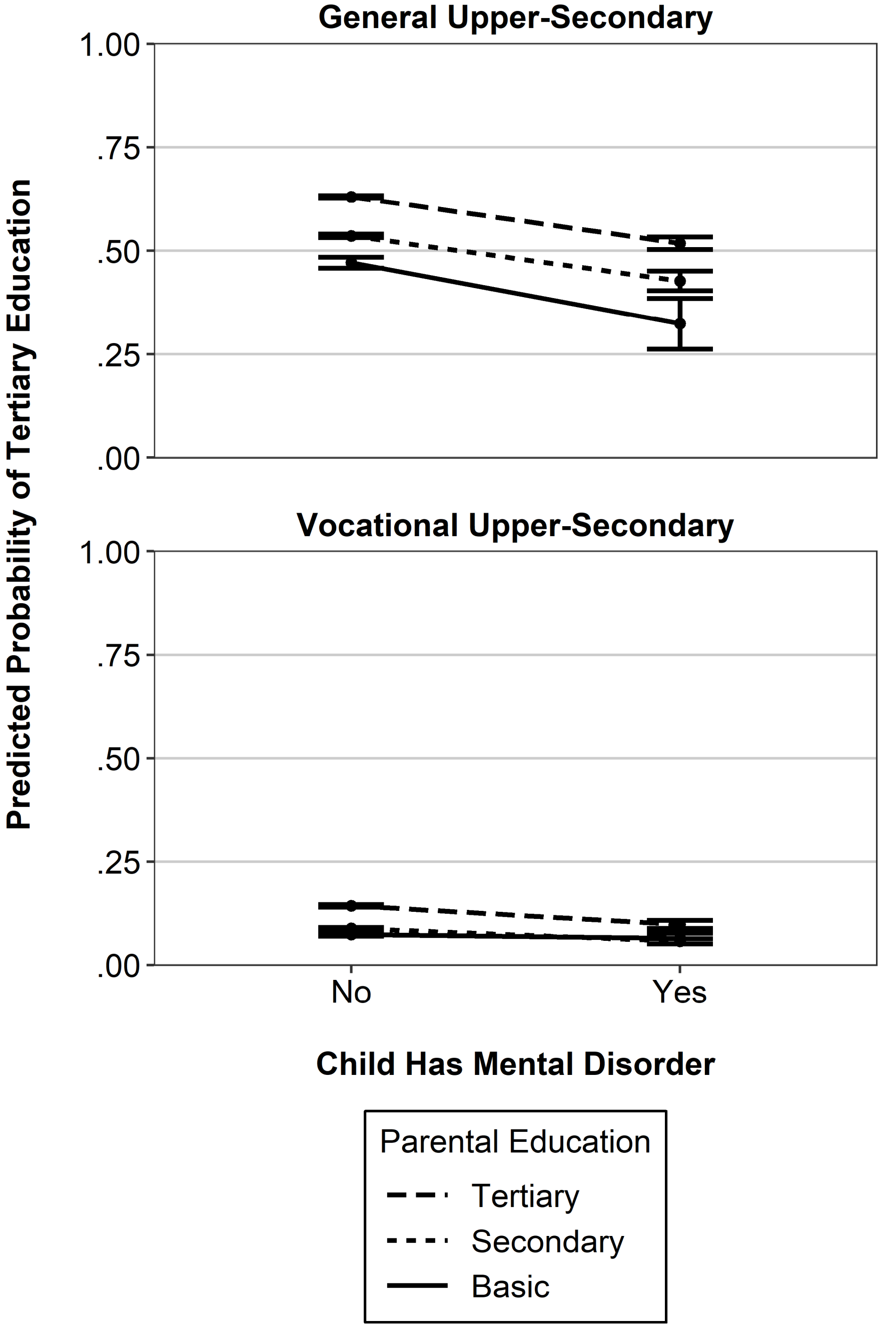

Supplement: Supplementary file 1 — (PDF 254 kb) [file 13524_2020_919_MOESM1_ESM.pdf]
